# Experimentally induced distraction impacts cognitive but not emotional processes in think-aloud cognitive assessment

**DOI:** 10.3389/fpsyg.2014.00474

**Published:** 2014-05-20

**Authors:** Kean J. Hsu, Kalina N. Babeva, Michelle C. Feng, Justin F. Hummer, Gerald C. Davison

**Affiliations:** Laboratory for Cognitive Studies in Clinical Psychology, Department of Psychology, University of Southern CaliforniaLos Angeles, CA, USA

**Keywords:** cognitive assessment, think-aloud, distraction, emotion, Articulated Thoughts in Simulated Situations

## Abstract

Studies have examined the impact of distraction on basic task performance (e.g., working memory, motor responses), yet research is lacking regarding its impact in the domain of think-aloud cognitive assessment, where the threat to assessment validity is high. The Articulated Thoughts in Simulated Situations think-aloud cognitive assessment paradigm was employed to address this issue. Participants listened to scenarios under three conditions (i.e., while answering trivia questions, playing a visual puzzle game, or with no experimental distractor). Their articulated thoughts were then content-analyzed both by the Linguistic Inquiry and Word Count (LIWC) program and by content analysis of emotion and cognitive processes conducted by trained coders. Distraction did not impact indices of emotion but did affect cognitive processes. Specifically, with the LIWC system, the trivia questions distraction condition resulted in significantly higher proportions of insight and causal words, and higher frequencies of non-fluencies (e.g., “uh” or “umm”) and filler words (e.g., “like” or “you know”). Coder-rated content analysis found more disengagement and more misunderstanding particularly in the trivia questions distraction condition. A better understanding of how distraction disrupts the amount and type of cognitive engagement holds important implications for future studies employing cognitive assessment methods.

## Introduction

Given the limited capacity of human cognition, engaging in any non-automatic task (including any type of cognitive-affective assessment) requires that attention be selectively focused on relevant information while irrelevant information is ignored. Distractors are frequently thought of as external sensory stimuli (e.g., sights or sounds), but they may also be internal processes (e.g., one's mood states, cognitions, physical sensations). Distraction, also known as task disengagement, represents a potential threat to the reliability and validity of any assessment procedure by interfering with an individual's ability to complete a task at peak performance. Although the detrimental effects of distraction on a variety of more basic cognitive tasks (e.g., visuo-spatial and working memory tasks) are well known (e.g., Lavie, 2005; Tremblay et al., 2005), studies explicitly examining the effects of distraction on engagement in cognitive-affective think-aloud paradigms are notably absent.

Cognitive-behavioral theory posits that the manner in which individuals interpret the world around them influences their behavioral and emotional responses. Acknowledging that thoughts are central to intra and interpersonal functioning as well as to psychopathology, psychologists have used a variety of questionnaires and assessment methods to capture cognitions and emotions. One such cognitive-emotional assessment approach is the Articulated Thoughts in Simulated Situations (ATSS) experimental paradigm. The term “paradigm” rather than “instrument” or “method” reflects that the ATSS is a basic approach to cognitive-emotional assessment and not a specific assessment technique or instrument. The ATSS paradigm (Davison et al., 1983) was introduced as a way to access complex cognition and emotion in experimenter-controlled situations of considerable complexity. This think-aloud paradigm involves the research participant being asked to imagine that he/she is part of an audio-recorded situation and to verbalize his/her thoughts and emotional reactions periodically during pauses in the vignette. Investigators can tailor the paradigm's parameters to suit particular experimental and clinical demands and preferences. As a result, the ATSS has been utilized in diverse areas of inquiry such as anger and aggression, interpersonal bias, psychotherapy process, eating disorders, alcohol use, maintenance of smoking cessation, depression, and social anxiety (for further detail, see reviews by Davison et al., 1997; Zanov and Davison, 2010).

The assessment of emotions and thoughts is qualitatively different from the assessment of more basic cognitive abilities. In particular, many cognitive ability tasks employ objective criteria such as response accuracy and speed to assess performance. However, subjective responses involving self-reported thoughts and feelings in the domain of cognitive-emotional assessment cannot be judged by the same performance-driven criteria. While it is necessary to exercise caution in drawing direct comparisons between the hypothesized effects of distraction in both cognitive domains, we also recognize the dearth of research into the effects of distraction on cognitive-affective assessments. Thus, the current study draws from select research examining the effect of distraction on assessments of cognitive abilities to examine the effects of distraction on the think-aloud cognitive-affective assessment paradigm known as the ATSS.

Research employing basic cognitive tasks reveals that, rather than being uniform across tasks, the impact of distraction varies based on the characteristics of the task and of the distractor. Specifically, the modality, perceptual and cognitive load of the task, as well as the modality and salience of the distractor have been found to be of importance (for a review, see Lavie, 2005). For example, a main determinant of the amount of interference a distractor has on task performance is the level of cognitive load (or difficulty) of a task. According to Lavie et al. (2004), if a target task places a high load on working memory, fewer cognitive resources are left to maintain selective attention and thus mind-wandering in response to task-irrelevant stimuli becomes more likely. This load theory of cognitive control suggests that the more the distractor competes with goal-directed behavior, the harder it is to maintain goal-relevant priorities (Lavie et al., 2004). Thus, if one is engaged in a complex cognitive task that is taxing to executive functioning, it becomes more difficult to sustain focus and ignore distractors. Indeed, studies employing paradigms that require switching between two tasks or performing two tasks simultaneously (such as a manual bolt screwing task and random letter generation task performed simultaneously; e.g., Bourke, 1996; Monsell, 2003) document performance decrement in response accuracy and speed as compared to single task performance. Applied to think-aloud cognitive-affective assessment, this theory suggests that high-load distractors (e.g., multi-component tasks) may impact responding by slowing down performance (i.e., limiting the amount of articulated thoughts and feelings) and increasing confusion and misunderstanding of the presented scenarios. In turn, slowed performance and mental confusion regarding information being presented could negatively impact the quality of participants' responses and subsequent conclusions drawn from the data, without the experimenter knowing that such threats to validity have taken place.

Studies that do not specifically examine task load suggest that shared input modality (e.g., visual or auditory) between simultaneous tasks increases interference between the tasks. For example, simultaneous presentation of inputs from the same modality (visual or auditory) produces more task interference than the simultaneous presentation of inputs from different modalities (Duncan et al., 1997). In order to not overburden participants by making task performance extremely difficult, the current study uses visual presentation of distractors during the ATSS, the input for which is auditory.

Semantic similarity of the information presented concurrently also increases interference (Hirst and Kalmar, 1987). For example, during a dichotomous listening task, participants demonstrated greater difficulty when they received the same type of input (letters or numbers) in both ears than when it was different (letters and numbers simultaneously). Previous research also suggests that auditory (spoken) and visual (written) presentation of semantic information activates some overlapping brain regions (e.g., Jobard et al., 2007; Buchweitz et al., 2009). That is, hearing and reading words activate similar neuronal pathways. This finding implies that a visual, semantic distractor is more likely to interfere with an open-ended think-aloud task than a visual, non-semantic distractor. The current study therefore includes both a semantic and non-semantic distractor.

The aforementioned studies all utilized tasks that do not explicitly involve emotion-provoking stimuli. However, emotion has important consequences for attention. Emotional information (usually negatively-valenced) interferes with and frequently takes precedence over neutral information presented simultaneously (e.g., Hartikainen et al., 2000; Öhman et al., 2001; Calvo et al., 2007). In situations that place constraints on attentional resources, attention is likely to be drawn toward emotional items, especially ones that may represent a threat (Mathews and Mackintosh, 1998), that have personal significance (Moray, 1959; Mogg and Bradley, 1998), or that are congruent with one's mood state (Yovel and Mineka, 2005; Salters-Pedneault et al., 2007). Furthermore, studies suggest that processing of emotional information may happen automatically, bypassing more effortful cognitive pathways (Zajonc, 1980). Consequently, processing self-relevant, emotion-laden information (such as a personally-relevant scenario eliciting negative emotion) may take precedence over other simultaneously occurring tasks.

The current study seeks to examine the effects of task disengagement on responding to the ATSS paradigm by experimentally inducing distraction. To test the effects of distraction, participants were asked to verbalize their thoughts and feelings in response to three commonly used types of hypothetical scenarios in the ATSS literature (i.e., a neutral, an anxiety-provoking, and an anger-provoking scenario) in one of two experimentally manipulated distraction conditions: (1) while playing Tetris or (2) while answering trivia questions. A third condition involved no experimentally-induced distractor. The distractors were chosen to mimic two main types of distraction that are most likely to occur while a participant is engaging in a cognitive-emotional assessment task. Playing Tetris represents an exogenous, non-verbal distractor that calls for a motor response (i.e., similar to playing a non-verbal game on one's cell phone or interacting with an object in the environment). Answering trivia questions is a endogenous verbal distractor that places a load on working memory and triggers thoughts relevant (or not) to the content of the trivia question (i.e., mimicking participants' engagement in a verbal task such as reading a text message, checking social media updates, etc.).

Based on the reviewed literature, the following hypotheses were generated. First, given that negatively-valenced, self-relevant information captures attention and given extensive evidence from over four dozen ATSS experiments (see review by Zanov and Davison, 2010), we expected that individuals in all conditions would be able to engage in the task and produce emotionally-congruent verbalizations (i.e., anger in the anger-provoking scenario and anxiety/worry in the anxiety-provoking scenario). In other words, no differences were expected in terms of the degree of articulated negative emotions (namely, anger, and anxiety) in distracted vs. non-distracted individuals across all three ATSS scenarios. However, considering the high cognitive load of the ATSS paradigm and the limited processing capacity of human cognition, we also expected the experimental distractions to detrimentally impact certain types of performance. We hypothesized that this interference would manifest itself in higher levels of articulated distraction, disengagement, and misunderstanding of the scenarios. Furthermore, given the semantic nature of the ATSS, we also expected that the semantic distractor (i.e., reading trivia questions) would produce more interference with respect to these parameters than the non-semantic distractor (i.e., playing Tetris). Lastly, as studies have shown that people may be less affected by distractors if they are focusing on an emotional task, we hypothesized that individuals would display more articulated distraction, disengagement, and misunderstanding of the scenarios in the neutral scenario than in the anger and anxiety scenarios.

## Materials and methods

### Participants

Participants were 102 students recruited from the psychology subject pool at the University of Southern California and received course credit for their participation in the study. Each participant attended a 1-h lab-based research session, during which they participated in the ATSS paradigm (see below; Davison et al., 1997). Prior to arrival, participants were randomized into three conditions: a Tetris distraction (where participants played a computerized Tetris game while listening to the audiotaped scenario), a Trivia distraction (where participants were required to read to themselves and then answer aloud as quickly as possible a trivia question with three multiple choice answers while listening to the audiotaped scenario), and a control condition (normal participation in the ATSS paradigm). Scenario order was counter-balanced across participants (for a total of 18 randomizations: 3 distraction conditions X 6 counter-balanced scenario orders). Thirty-four participants (6 male) were randomized into the Tetris distraction, 28 participants (6 male) were randomized into the Trivia distraction, and 40 participants (5 male) were randomized into the control condition. There were no significant differences in age, ethnicity, or education across conditions (all *p* > 0.05). The study protocol was approved by the local Human Subjects Institutional Review Board and all participants provided informed consent prior to participation.

### Measures and procedure

#### Procedure

Instructions for participating in the ATSS paradigm were presented both by audio and in written format simultaneously via desktop PC. To help improve the quality of responses elicited from the participant by the stimulus recordings, a practice scenario was used during which the experimenter provided feedback about the participant's think-aloud performance (e.g., “Try to fill the whole thirty seconds of silence” and “Please discuss both your thoughts and your feelings as you imagine yourself in this scenario right now”) to encourage maximal involvement in the procedure. After completing the ATSS paradigm, participants were debriefed by a research assistant and assessed for self-reported distraction and attentiveness over the course of the study.

#### Articulated thoughts in simulated situations

Three audio scenarios were employed. Each scenario consisted of seven segments lasting approximately 20 s. After each individual segment, a tone signaled the beginning of a 30-s silence during which participants verbalized their thoughts and emotions as if they were in that scenario at that immediate moment in time. Distractions (as described above) were presented during the stimulus section but not during the 30-s period of silence during which participants articulated their thoughts and feelings. The stimulus recordings consisted of the following:

***Auto mechanic garage tape (anger-inducing scenario)***. The participant was asked to imagine that he/she had left work early to pick up his/her car at the mechanic. A female secretary greeted the participant and notified the manager that the participant had arrived for his/her car. During the course of the scenario as narrated throughout seven segments, the participant overheard the manager and mechanic discuss the status of the participant's car, which was not ready for pick up and in need of new parts. The manager suggested cheating the participant by charging more for parts as well as asking for more time to repair the car.

***Midterm tape (anxiety-inducing scenario)***. The participant was asked to imagine that it was the day of his/her psychology midterm and that he/she was sitting outside the room before the exam, overhearing two fellow students discuss the upcoming test. The two speakers discussed the extremely difficult nature of the course as well as last-minute changes to the test format that made the exam more challenging and intimidating. During the course of the tape, the participant found out that they had not received prior notice of exam changes and a review session through an electronic bulletin board.

***Cafeteria tape (neutral scenario)***. The participant was asked to imagine that he/she was waiting in line for lunch and overhearing two students in line discuss plans for the upcoming weekend.

## Results

### Trained human content analysis

Three undergraduate research assistants were trained on a total of five coding categories, including two emotional codes: (1) anger, hostility, and aggression; and (2) distress/anxiety/worry; and three distraction-specific codes: (3) overt distraction; (4) misunderstanding, and (5) disengagement. All coding categories were scored on a four-point (0–3) scale, considering both the amount and quality of expressions utilized by participants. Scores for each category were summed across segments for each scenario. Coding meetings were held weekly when coding initial participants to discuss coding challenges and inconsistencies. After this intensive training period, coders met approximately once a month to reduce coder drift. Coders showed very high absolute agreement for all codes: Anger intraclass correlation coefficient (ICC) = 0.978, Distress/Anxiety ICC = 0.980, Overt Distraction ICC = 0.996, Misunderstanding ICC = 0.983, Disengagement ICC = 0.954.

Anger, hostility, and aggression (AHA) captured statements and feelings of strong displeasure, antagonism, and/or “angry annoyance.” Distress/anxiety/worry referred to feelings of worry and nervousness. Overt distraction focused on direct statements of distraction or confusion by the participant. Misunderstanding gauged distraction based on *accuracy* of verbalizations to the scenario; this code required coders to attend to segment content and have a sense of what would and would not be appropriate/relevant (e.g., expressing anxiety about a psychology essay due, rather than a psychology midterm, which was the theme of one of the stimulus tapes). Disengagement was a judgment of how interested the participant was in the segment and how well they participated/worked with the material; this category reflects verbalizations that were disjointed, unfocused, jumbled, bored, or reflected apathy.

### Linguistic inquiry and word count coding

Transcripts for each participant were also coded through the Linguistic Inquiry and Word Count software program (LIWC; Pennebaker et al., 2007). LIWC has been used extensively to code written statements and verbal transcripts (for review, see Tausczik and Pennebaker, 2010). The LIWC program codes verbal material by identifying the percentage of words fitting into a particular code category. For the purposes of this study and given the nature of the scenarios presented, we used the LIWC emotional codes of anger (e.g., hate, kill, annoyed), and anxiety (e.g., worried, fearful, nervous) to measure emotional expression.

To operationalize distraction through the LIWC program, we also examined cognitive process codes and spoken category codes. Cognitive process codes identify different facets of thinking, including insight-oriented words (e.g., think, know, consider) and causation words (e.g., because, effect, hence), and may reflect processes of cognition that differ between experimentally distracted and non-distracted individuals. The spoken category codes used here were non-fluencies (e.g., errr, hmmm, umm) and fillers (e.g., blah, I mean, you know) and have been suggested to represent disorganized thinking process (Tausczik and Pennebaker, 2010). We also considered the total number of words used by each participant as an indicator of distraction.

Due to the non-normal nature of the main outcomes, non-parametric (i.e., Kruskal-Wallis) One-Way ANOVAs were conducted for each hypothesis, using distraction type or scenario type as the grouping variable. Non-parametric pairwise comparisons were conducted for outcomes significantly differing across all groups in order to determine pairwise differences between individual groups with a two-tailed Bonferroni-corrected alpha level of 0.05. All analyses were conducted using SAS software (SAS version 9.2, SAS Institute, Cary, NC).

### Manipulation check: distraction

Distractor task scores were not analyzed due to the nature of the distractor tasks. The trivia questions were selected for their difficult and thought provoking nature; as a consequence, low performance is not indicative of low levels of distraction. The tetris distractor task was rate limited so that blocks fell at a constant pace. As a result, variability in score was also reduced and not necessarily indicative of engagement with the distractor. Consequently, in order to verify the impact of the experimentally-induced distractions on our think-aloud cognitive assessment paradigm, we examined both overt distraction (coded by trained research assistants) as well as self-reported distraction and attentiveness between the three conditions. Overt distraction differed significantly by distraction condition, *H*_(2)_ = 7.19, *p* = 0.028. Pairwise comparison found that overt distraction was highest in the trivia condition, with significant differences between trivia and control, and marginal differences between trivia and tetris (and thus no statistically significant difference between the control and tetris conditions).

This pattern of results also was found in self-reported distraction, with significant differences between conditions, *F*_(2, 70)_ = 8.12, *p* < 0.001 (self-reported distraction and attentiveness were normally distributed, hence the use of the F-statistic). Again, pairwise comparisons found that distraction was highest in the trivia condition but no statistically significant difference between the control and tetris conditions was found. Self-reported amount of attention paid during the course of the study also significantly differed between groups, *F*_(2, 69)_ = 12.27, *p* < 0.001. However, pairwise comparisons found that individuals in the control condition were able to pay significantly more attention than in the tetris and trivia conditions. These analyses indicate that, as predicted, participants in the trivia condition felt greater distraction in their condition relative to participants in the other two conditions.

### Manipulation check: emotional congruence

The degree of anger and anxiety differed significantly by scenario types in the proportion of expressed anger, *H*_(2)_ = 70.99, *p* < 0.001 and anxiety, *H*_(2)_ = 82.71, *p* < 0.001. (Table 1 here) Table 1 displays the mean proportion of LIWC emotion codes and rated emotion codes by scenario type along with the overall and pairwise comparison analyses. Pairwise comparison revealed that the anger scenario elicited the most anger of all three scenarios, with no significant differences in anger between the neutral and anxiety scenarios. Similarly, the anxiety scenario elicited the most anxiety of all three scenarios; the anger and neutral scenarios did not differ significantly in proportion of anxiety words used. These results confirm that the emotional scenarios were successful in eliciting the desired emotional response from participants.

**Table 1:** **Mean (Standard Deviation) LIWC emotion code scores^a^ and total coder-rated emotion scores for the anger (ANG), anxiety (ANX), and neutral (NEU) ATSS scenarios**.

**Emotion code**	**Anger scenario (ANG)**	**Anxiety scenario (ANX)**	**Neutral scenario (NEU)**	**Scenario main effect (Kruskal-Wallis non-parametric ANOVA)**	**Non-parametric pairwise comparisons**
LIWC anger	1.36 (1.18)	0.49 (0.67)	0.40 (0.48)	*H*_(2)_ = 70.99, *p* < 0.001	ANG > ANX, NEU
LIWC anxiety	0.49 (0.43)	1.57 (1.52)	0.39 (0.46)	*H*_(2)_ = 82.71, *p* < 0.001	ANX > ANG, NEU
Rated anger	8.36 (4.01)	1.21 (1.53)	2.21 (2.58)	*H*_(2)_ = 70.99, *p* < 0.001	ANG > ANX, NEU
Rated anxiety	1.31 (1.59)	6.98 (4.60)	1.40 (2.04)	*H*_(2)_ = 82.71, *p* < 0.001	ANX > ANG, NEU

a *LIWC scores are in percentage of words out of the entire participant's transcript*.

### Word count

The number of words used across all scenarios significantly differed across conditions, *H*_(2)_ = 6.36, *p* = 0.042; mean words (*SD*) used by condition per scenario were 495.70 (118.66) for the control condition, 466.28 (109.49) for the trivia condition, and 466.26 (119.06) for the tetris condition. Pairwise comparisons indicated that participants in the control condition used significantly more words than participants in the two distraction conditions, which did not significantly differ from one another. Consequently, we used proportion of words in each LIWC category as our main outcome rather than number of words, given the significant differences in word count between conditions.

### Impact of distraction on verbalized emotion

Using data from the LIWC program, no significant differences were found among the three conditions of distraction (tetris, trivia, no distraction) for verbalized anxiety, *H*_(2)_ = 2.53, *p* = 0.282, or anger, *H*_(2)_ = 0.39, *p* = 0.824 across all scenarios. Similarly, no significant differences were found among the three conditions for coder-rated anger, *H*_(2)_ = 3.46, *p* = 0.178 or anxiety, *H*_(2)_ = 0.013, *p* = 0.994. These results suggest that level of distraction did not affect the emotional involvement of participants in the three scenarios.

### Impact of distraction on cognitive engagement

(Table 2 here) To examine cognitive engagement in the scenarios, analyses were conducted to test if there were differences between condition on LIWC codes for insight and causal words, as well as the LIWC codes of fillers and non-fluencies. Condition was significantly associated with proportion of insight words used, *H*_(2)_ = 6.97, *p* = 0.031, and with proportion of causal words used, *H*_(2)_ = 15.12, *p* < 0.001. Table 2 displays the mean proportion of cognitive process and spoken category codes by condition along with the overall and pairwise comparison analyses. Pairwise comparisons showed that participants in the trivia questions distraction produced a significantly higher proportion of insight and causal words than participants in the tetris and non-distraction conditions, but that there was no difference between the latter two conditions in these two codes. These results suggest that the trivia condition significantly impacted cognitive engagement in the scenarios.

**Table 2:** **Mean (Standard Deviation) LIWC cognitive processing and spoken category code Scores^a^ and total coder-rated disengagement and misunderstanding for the trivia question (TRV), tetris (TET), and control distraction (CTL) conditions**.

**Code**	**Trivia question distraction (TRV)**	**Tetris distraction (TET)**	**Control condition (CTL)**	**Distraction main effect (Kruskal-Wallis non-parametric ANOVA)**	**Non-parametric pairwise comparisons**
Insight words	3.83 (2.21)	3.20 (1.49)	3.15 (1.48)	*H*_(2)_ = 6.97, *p* = 0.031	TRV > TET, CTL
Causal words	1.91 (0.91)	1.53 (0.92)	1.50 (0.81)	*H*_(2)_ = 15.12, *p* < 0.001	TRV > TET, CTL
Non-fluencies	2.95 (2.15)	1.79 (1.51)	2.02 (1.46)	*H*_(2)_ = 17.17, *p* < 0.001	TRV > TET, CTL
Fillers	1.67 (1.40)	1.34 (1.48)	1.73 (1.54)	*H*_(2)_ = 8.99, *p* = 0.011	CTL > TET, CTL = TRV
Disengagement	7.04 (4.85)	5.93 (4.18)	5.17 (3.70)	*H*_(2)_ = 7.08, *p* = 0.029	TRV > TET, TET = CTL
Misunderstanding	1.69 (2.27)	0.94 (2.14)	0.60 (1.22)	*H*_(2)_ = 15.20, *p* < 0.001	TRV > TET, CTL
Mean words per scenario	466.28 (109.49)	466.26 (119.06)	495.70 (118.66)	*H*_(2)_ = 6.36, *p* = 0.042	CTL > TET, TRV

a *LIWC scores are in percentage of words out of the entire participant's transcript*.

Examination of the spoken category codes indicated that condition was significantly associated with use of non-fluencies, *H*_(2)_ = 17.17, *p* < 0.001 and with fillers, *H*_(2)_ = 8.99, *p* = 0.011. Pairwise comparisons again showed that individuals in the trivia distraction condition verbalized a higher proportion of non-fluencies than did participants in both the tetris and non-distraction conditions and that there were no significant differences between the tetris and non-distraction conditions. Pairwise comparisons for the impact of distraction condition on filler words used indicated that the control condition elicited significantly more fillers than the tetris condition, with no difference between the trivia and control conditions. These analyses imply that the trivia condition may result in more disorganized thought than the tetris or non-distraction conditions.

Distraction condition was also associated with significant differences in disengagement, *H*_(2)_ = 7.08, *p* = 0.029 and in misunderstanding, *H*_(2)_ = 15.20, *p* < 0.001. Pairwise comparisons found that individuals in the trivia distraction exhibited more disengagement than participants in the control condition, with no significant difference between the tetris and control condition. Individuals in the trivia distraction condition showed significantly higher misunderstanding than both the tetris and control condition participants. These results indicate that participants in the trivia condition display less engagement in the think-aloud assessment than participants in the other two conditions.

### Impact of scenario type on cognitive engagement

Scenario type was also related to proportion of insight words utilized, *H*_(2)_ = 51.62, *p* < 0.001, as well as causal words, *H*_(2)_ = 18.33, *p* < 0.001 but not non-fluencies, *H*_(2)_ = 5.17, *p* = 0.078 or filler words, *H*_(2)_ = 4.93, *p* = 0.085. Pairwise comparisons indicate that the anxiety scenario provoked significantly greater use of insight words than the anger and neutral scenarios. The neutral scenario also was associated with greater use of causal words compared to the anger and anxiety scenarios. These results suggest that the different scenarios may elicit different patterns of cognitive engagement.

Scenario type was also significantly associated with coder-rated misunderstanding, *H*_(2)_ = 16.71, *p* < 0.001 but not disengagement, *H*_(2)_ = 1.38, *p* = 0.502. Pairwise comparisons found that the anger scenario was associated with significantly greater misunderstanding compared to the anxiety and neutral scenarios, with no significant difference between the anxiety and neutral scenarios. This result may indicate that understanding the anger scenario is more difficult than understanding the anxiety and neutral scenarios.

## Discussion

While the impact of distraction on task performance has been well studied, its impact on cognitive assessment is less well-documented, particularly regarding think-aloud assessment, and emotion-provoking stimuli. This empirical neglect is surprising given that task disengagement has often been raised by critics as a threat to assessment validity, though primarily directed toward think-aloud methods. The current study examined this issue by experimentally manipulating distraction for individuals participating in a think-aloud assessment method known as the Articulated Thoughts in Simulated Situations paradigm.

The first aim was to assess the impact of distraction on verbalized emotions in response to three scenarios—one designed to induce anger, one to induce anxiety, and one to be relatively emotion-neutral. The scenarios were found to elicit the intended emotions among all individuals (e.g., the anger scenario being associated with the largest proportion of anger expressions and coder-rated anger), and distraction did not affect the proportion of emotions expressed. That is, think-aloud cognitive assessment of emotion in this study was not significantly impacted by task disengagement, regardless of distractor modality. This finding supports the notion that emotionally salient stimuli are robust in the context of distraction and take attentional precedence over neutral stimuli (Öhman et al., 2001; Calvo et al., 2007). Our findings also support research by Zajonc (1980) that affective judgments and reactions have an innate quality and are not necessarily contingent upon extensive cognitive processing (or even recognition of the stimulus). Affective reactions may thus be less disrupted by distractors that interfere with cognitive encoding. In other words, individuals may experience a fairly automatic emotional response to a stimulus without having extensively encoded the characteristics of the stimulus (as might be the case in the presence of a distractor).

This phenomenon is well-documented in studies of fear reactions that occur automatically and do not depend on higher-level cortical pathways (for an overview, see LeDoux, 2003; Vuilleumier, 2005) as well as in the domain of impression formation, where individuals have been found to quickly and automatically form judgments about a person after very brief exposure to his/her face without effortful encoding of his/her characteristics (e.g., Willis and Todorov, 2006; Todorov et al., 2009). As a result, individuals may have an emotional reaction, especially in response to threatening stimuli, without having to accurately perceive or consciously think about the stimuli. Thus, despite being under conditions of distraction, participants in this study may have been able to react with anger and anxiety to the presented scenarios, as accurate perception and sophisticated cognitive processing of all of the details of the situation were not necessary in order to have an affective response.

Furthermore, affective information is readily conveyed via non-verbal vocal cues, such as the tone of a speaker's voice (e.g., Argyle et al., 1970). Given that all three of the experimental scenarios used in this study included overheard conversations between two speakers, affective information is likely to have been easily gleaned by participants from non-verbal vocal cues even when the processing of the verbal content of the overheard conversations was disrupted. Indeed, participants are capable of identifying the emotional valence of speech even when its content is digitally masked (e.g., Scherer et al., 1972). Thus, our participants are likely to have gleaned and reacted to emotional information from vocal cues even in the presence of distraction.

Results of the current study indicate that emotion-focused cognitive assessment, at least in the case of the think-aloud paradigm used in this study, appears not to be significantly impacted by wandering attention during stimulus presentation immediately prior to assessment. However, emotional distractors have been found to negatively impact performance on cognitive (e.g., working memory) tasks (Dolcos and McCarthy, 2006; Dolcos et al., 2008; Denkova et al., 2010), due in part to disruption in brain regions involved in attentional processes (e.g., the dorsolateral prefrontal cortex; Dolcos and McCarthy, 2006). Affectively-based distractions might have a stronger impact on cognitive and/or affective assessments, particularly given the strength of emotional distractors relative to non-emotional distractors (Dolcos et al., 2008). Thus, the extent to which emotion-focused cognitive assessment is disrupted by emotional or valenced distractions deserves further investigation and needs to include the use of different cognitive tasks, as well as various cognitive and/or affective paradigms.

The second aim of this study was to examine the impact of task disengagement on cognitive processes by considering differences in the use of causal words, insight-oriented words, filler words, and non-fluencies, as well as coder-rated disengagement and misunderstanding. The hypotheses were partially supported. Distraction appeared to significantly affect the organization of participants' thought, though this effect was confined primarily to the trivia distraction condition. Unexpectedly, distracted individuals used more of these types of mental organization words. Tausczik and Pennebaker (2010) put forth that causal and insight words “can suggest the active process of reappraisal” (p. 36); in this study, causal and insight words may have been utilized to re-process some of the information the participant was able to hear in order to form a more coherent and cohesive narrative based on limited information. The notion of narrative formation through greater employment of causal words is supported by Boals and Klein (2005), who posited the use of causal words as indicative of an individual processing thoughts about an event and attempting to create “causal connections” (p. 263). As distracted participants in the ATSS paradigm might not have access to complete portions of the audio stimuli, they may be forced to process chunks of the scenario during their think-aloud response, organizing information as it is recalled from short-term memory.

The trivia questions distraction condition also resulted in greater proportions of non-fluencies than the other conditions. Use of non-fluencies like “umm” and “uhh” has been associated with increased cognitive load (Smith and Clark, 1993; Sporer and Schwandt, 2006) and has been found to occur more when fabricating information (i.e., lying; Vrij et al., 2011). These behaviors are in line with reorienting from distraction in an assessment situation; some individuals may try to process the limited information when not distracted in the context of the overall assessment (e.g., scenario stimuli), while other individuals might attempt to fabricate plausible responses to the assessment prompts based on their understanding of the content to that point in the assessment.

In both of these possible situations during think-aloud assessment, a large amount of cognitive resources is required to simultaneously process information or fabricate responses while also responding aloud to the prompt with their thoughts and feelings. Based on this premise, greater cognitive resources ought to be associated with resiliency in the face of distraction. Meys and Sanderson (2013) found that individuals with better cognitive resources (in the form of greater working memory capacity) were more resistant to distraction during a running arithmetic task. Working memory may represent an individual difference factor that buffers against distraction across assessment paradigms and should be examined in future studies on the impact of distraction on task performance. In addition, it is possible that the ATSS procedure is relatively immune to these distractors.

While the trivia distraction led to significant differences on these primary outcomes, the Tetris distraction manipulation generally did not differ significantly from the control condition on these outcomes. As the trivia distractor was a verbally based manipulation, the overlap in the linguistic domain between the distraction and the stimuli may have resulted in competing processes and greater interference. Indeed, in a study of memory for visually presented digits with audio presentation of irrelevant digit, word, and non-word distractors, Salamé and Baddeley (1982) showed that recall accuracy declined the most in the presence of digits and phonologically similar word distractors.

Taken together, the findings of the current study support the idea that while distractors like phone-based video games might not significantly impact performance or engagement in self-report assessments, other types of distractors like texting may harm the fidelity of responses acquired from respondents, regardless of assessment modality (e.g., questionnaire, interview, think-aloud, etc.). This area of research is largely unexplored. Previous research on distraction has almost exclusively studied the impact of distraction on tasks whose outcomes are typically based on some element of time. Only two studies to our knowledge have looked at distraction on assessments that used outcomes that were more qualitative or less time-dependent in nature. Dixon and Salley (2006) utilized a fast mapping (i.e., word-learning) protocol whereby children were presented five objects, four that were known exemplars and one that was a novel object. After given some time to explore the objects, children were asked to identify objects when provided the name. Novel labels (e.g., “dax” or “noop”) were used for the novel exemplar and repeated in order to improve the learning process. A second tray was presented thereafter, with four known objects and two novel objects, one taxonomically related and one completely unrelated object (i.e., a foil). Two distraction conditions were utilized: one condition that involved increasing cognitive load via the addition of an additional familiar object during the learning phase, and another that involved sudden distractions in the environment (e.g., a stranger walking into the room and reading a book, or a mechanical toy being operated in view of the child). Both distraction conditions impaired novel word acquisition. In another study, Thrift (2012) used an information visualization task to examine the impact of environmental distraction on the ability to accurately discern patterns and trends in data presented to participants. Subjecting participants to a noisy environment resulted in decreased task performance accuracy. Both of these studies support the current findings that distraction can significantly impact performance in the context of assessment.

### Limitations

The current study utilized a sample composed of college students. It is unclear the extent to which these results generalize to other age groups and educational backgrounds, particularly given that cognitive functioning (e.g., processing speed and working memory) and thus the impact of distraction may change over the lifespan.

Most studies examining the impact of distraction on task performance use distractors that have correct responses, allowing for response accuracy to be used as a measure of distraction. The present research did not have a similar check in place due to the nature of the distraction conditions (e.g., trivia questions were selected for their difficulty and thought-provoking nature, meaning that most individuals did not select the right answer). However, the distraction manipulations were designed to be “heavy-handed” enough to test the upper limits, as it were, of the effects of distraction in a think-aloud assessment method. They were simultaneously designed to not overly burdensome on the participant, to avoid floor effects in performance. Results suggest that the modality of distraction may be critical in determining whether a distractor is inconsequential or impairing for individuals engaged in an assessment.

Lastly, the non-parametric nature of the data, in general, and consequent use of non-parametric analyses like the Kruskal-Wallis One-Way ANOVA, specifically, precludes the reporting of effect size coefficients in these analyses. In addition, due to the non-normality of the data, the study is likely underpowered to adequately detect interaction effects. Further experimental studies with larger sample sizes are suggested to test for interaction effects with appropriate power.

### Strengths, conclusions, and future directions

The current study is among the first to examine the impact of task disengagement on cognitive assessment. Studies of distraction have overwhelmingly focused on the impact of distraction on task performance. In addition, distraction was experimentally manipulated in this study, allowing for causal inferences based on our findings. Moreover, we used two methods of content-analysis for the responses provided to this think-aloud cognitive assessment paradigm, with a consistent pattern of results across both content-analyses, which strengthened the reliability of the results.

On the whole, one has little to no idea what the social and environmental context is when a participant responds to an online assessment. Less attended-to aspects of the testing environment (such as ambient temperature or type of background noise present) have been found to have significant impacts on engagement and performance (e.g., Hancock, 1984; Enander, 1987; Hancock and Warm, 1989) and are thus important to consider in assessment, where individuals seek to gain accurate information regarding an individual's current state in a particular domain of functioning.

We also found that the distraction conditions did not differ in terms of the proportion of emotional responses elicited, indicating that distraction did not significantly impact the level of emotional intensity/responsivity to the scenarios. This is an important finding, as it suggests that even in the context of distraction, intended emotional responses can be elicited and assessed in a valid manner. For future studies employing cognitive assessment methods, more systematic consideration of how distraction can and does disrupt cognitive engagement is necessary, whether in the form of additional measures of engagement or use of attention tracking devices such as eye tracking programs.

The present research indicates a need for more empirical attention to be directed toward understanding the impact of distraction on cognitive assessment given that these kinds of assessments are often administered without regard to participant environment or engagement. Distraction can negatively impact both timed and non-timed cognitive tasks (e.g., word-learning tasks; Dixon and Salley, 2006), suggesting that assessment, in all its forms, ought to attend more to the impact of environmental distractions. Ignoring or disregarding the possible impact of distractors on assessment validity becomes particularly concerning as more and more research involves online administration of measures, instruments, and tasks. Study participants may be engaged in a number of different extraneous tasks that may result in drawing attention away from the desired engagement in responding. As described above, these distractions may negatively impact the amount and type of cognitive engagement that takes place, thereby raising questions about validity in the various assessment approaches.

### Conflict of interest statement

The authors declare that the research was conducted in the absence of any commercial or financial relationships that could be construed as a potential conflict of interest.
